# Retraction for Saeed et al., “Parvulin 14 and Parvulin 17 Bind to HBx and cccDNA and Upregulate Hepatitis B Virus Replication from cccDNA to Virion in an HBx-Dependent Manner”

**DOI:** 10.1128/jvi.01187-25

**Published:** 2025-09-12

**Authors:** Umar Saeed, Jumi Kim, Zahra Zahid Piracha, Hyeonjoong Kwon, Jaesung Jung, Yong-Joon Chwae, Sun Park, Ho-Joon Shin, Kyongmin Kim

## RETRACTION

Volume 93, no. 6, e01840-18, 2019, https://doi.org/10.1128/jvi.01840-18. The authors hereby retract this article. Upon review, we realized that several immunoblot images and other data were reused within the article.

Had this issue been identified earlier, we could have addressed it promptly by referring to the original lab notebooks. However, more than 7 years have passed since the experiments were conducted. As a result, it is now extremely difficult to retrieve or verify the original experimental records.

Because of these unresolved concerns, we are retracting the article and regret any inconvenience caused to the readers.

